# Response to "Analysis of possible risk predictors in patients with coronavirus disease 2019: a retrospective cohort study"

**DOI:** 10.1590/1806-9282.20231418

**Published:** 2024-04-22

**Authors:** Marium Amjad

**Affiliations:** 1 Dow University of Health Sciences – Karachi, Pakistan.

Dear Editor,

I have read with great sincerity the article entitled "Analysis of possible risk predictors in patients with coronavirus disease 2019: a retrospective cohort study" by Beatriz Nienkotter et al^1^. It was a pleasure for me to read the concisely written article, and I congratulate the authors for their excellent efforts. I agree with the ultimate findings of the study that age older than 65 years and a lung involvement extension greater than 50% are predictors of a poor prognosis for coronavirus disease 2019 (COVID-19), as well as the need for high-flow oxygen therapy.

Based on varied research^2,3^, I agree that different clinical and computed tomography (CT) findings are associated with critical illness and a poor prognosis in patients with COVID-19. However, it seemed noteworthy to mention a few more points that would enhance the quality of this article and enrich its conclusion.

The retrospective sort of study has drawn numerous concerns due to the possibility of recall bias, which could be addressed if authors had included present cases of that time. The author could use a larger population to supplement the results. For instance, a 2020 prospective cohort study describes the characteristics of 5,279 patients with COVID-19 treated at a large quaternary academic health system^2^. Also, conducting a study at a particular location could create bias due to different socio-economic, health, and environmental conditions. As in this study, patients were all from a single geographical region and treated within a single health system; factors associated with poor prognosis might differ elsewhere. In the 2022 cohort study, patients were included in a multicenter international registry that had data from 31 centers in 7 countries^4^.

As it is entrenched that patients with a positive diagnosis of COVID-19 were included, laboratory confirmation of the cases could also be addressed. Numerous studies^2,4^ confirmed cases of COVID-19 using genetic sequencing or real-time reverse transcriptase polymerase chain reaction (RT-PCR). Additionally, the author should have analyzed further laboratory test findings, including blood urea nitrogen, serum creatinine, low-density lipoprotein levels (LDL), and liver function tests. A 2021 study concluded that elevated levels of aspartate aminotransferase (AST), creatinine, blood urea nitrogen, and bilirubin were significantly associated with unfavorable outcomes, and these parameters can be used to predict disease prognosis^5^. The author should also have briefed about different chronic conditions such as dyslipidemia, heart disease, chronic kidney disease, cancer, cerebrovascular disease, Parkinson's disease, and dementia since these comorbidities can influence mortality^6^. Dyslipidemia plays an important role in the pathological development of COVID-19. LDL levels are inversely correlated with disease severity, which could be a predictor of disease progress and poor prognosis^7^.

Although it was stated that pulmonary involvement at the first chest tomography was considered a risk factor for an unfavorable outcome, it can also be mentioned that pleural effusion and a higher CT score on the CT scan at the time of admission are imaging predictors of poor prognosis in COVID-19 patients^3^. Finally, these findings lead to the conclusion that different parameters can be used to predict disease prognosis. Monitoring disease progression from the early stages will help in reducing unfavorable outcomes and allow more targeted lines of treatment, leading to a reduction in the rate of mortality.

## ETHICS

The study was conducted in accordance with the Declaration of Helsinki and followed ethical standards.
